# Transcutaneous Spinal Stimulation Modulates Spinal Reflex Circuit Excitability in Persons with Spinal Cord Injury

**DOI:** 10.3390/biomedicines13092195

**Published:** 2025-09-08

**Authors:** Evan B. Sandler, Jennifer Ann Iddings, Karen Minassian, Edelle C. Field-Fote

**Affiliations:** 1 Crawford Research Institute, Shepherd Center, Atlanta, GA 30309, USA; evan.sandler@shepherd.org (E.B.S.); jennifer.iddings@shepherd.org (J.A.I.); 2 Center for Medical Physics and Biomedical Engineering, Medical University of Vienna, 1090 Vienna, Austria; karen.minassian@meduniwien.ac.at; 3 Division of Physical Therapy, Emory University School of Medicine, Atlanta, GA 30322, USA; 4 Program in Applied Physiology, Georgia Institute of Technology, Atlanta, GA 30332, USA

**Keywords:** spinal cord injury, transcutaneous spinal stimulation, paired-pulse depression, spasticity, noninvasive, afferent stimulation

## Abstract

**Background**: Transcutaneous spinal stimulation (TSS) is a noninvasive stimulation approach to modulate spinal reflex circuit excitability after spinal cord injury (SCI) Posterior root muscle (PRM) reflexes can be used to characterize the change in excitability of spinal reflex circuits after TSS; these responses are likely influenced by stimulus parameters. **Methods**: We compared PRM reflex responses to 3 TSS conditions: single-site continuous (SS-CONT), single-site burst (SS-BURST), and dual-site continuous (DS-CONT). Stimulation (frequency: 50 Hz, intensity: 80% soleus reflex threshold[RT]) was delivered for 30 min. The cathode was placed over the thoracic spine (T11–T12) and anodes placed paraumbilically; a second cathode over the lumbar spine (L1/2 or L2/3) was used for DS-CONT. PRM reflex responses in the soleus were elicited by paired 1 ms monophasic conditioning–test stimuli at a 50 ms interstimulus interval via the T11–12 cathode and paraumbilical anodes. Soleus PRM reflex indices included RT, response amplitude at 1.2xRT (RA_1.2xRT_), slope, area under the input–output curve (AUC). Paired-pulse indices were collected, including paired-pulse depression (PPD) and depression of the area under the curve (AUC_dep_). To assess the correlation between biomechanical and electrophysiologic measures of soleus spasticity, the ankle clonus drop test first drop excursion (FDE) was measured. All indices were measured at baseline and immediately post-intervention. **Results**: In whole-group analyses, PPD and AUC_dep_ were significantly decreased. Significant decreases in PPD and AUC_dep_ were identified only after the SS-CONT condition. No significant changes were identified in other PRM reflex indices after any of the 3 TSS conditions. No relationships between baseline FDE and any PRM reflex parameter were identified at baseline. **Conclusions**: With stimulation intensity of 80% soleus RT, modulation of targeted spinal reflex circuits was observed only in the SS-CONT condition when the response of the conditioning and test stimuli were considered. In addition, stretch-induced spasticity of the soleus may not be consistent with electrophysiologic testing.

## 1. Introduction

For individuals with spinal cord injury (SCI), spasticity has been described as “an over-reaction to every kind of input” [1], limiting function in activities of daily living and negatively impacting quality of life [2,3]. Reduction in descending inhibitory influences results in the dysregulation of local spinal circuits presenting as hyperexcitability of spinal motoneurons and spinal interneurons [4]. In preclinical models of SCI, maladaptive changes to serotonin receptors [5,6,7,8,9,10], disruption of chloride homeostasis [11,12,13,14], reduced primary afferent depolarization [15,16], and increased persistent inward currents [17,18] have been identified as promoting hyperreflexia. These mechanisms may individually or collectively contribute to augmented excitability of motoneurons. Activation of sensory afferents modulates these mechanisms associated with hyperreflexia/spasticity [6,19,20,21,22,23,24,25,26]. Transcutaneous spinal stimulation (TSS) is an accessible approach to achieve multi-segmental neuromodulation of spinal circuits through sensory afferent activation and, subsequently, spasticity reduction [27,28,29,30,31,32,33].

Electrophysiologic reflex assessments measure the integrity, excitability, and modulability of the neural pathways implicated in spasticity/hyperreflexia. Specifically, the H-reflex is commonly used to quantify excitability of the Ia monosynaptic spinal reflex pathway [34,35,36,37,38,39,40,41]. Posterior root-muscle (PRM) reflexes represent an analog to the H-reflex with the additional benefit of being elicitable in multiple lower extremity muscles bilaterally with a single stimulus [42]. Multiple indices of PRM reflex excitability differ in individuals with SCI compared to neurologically intact individuals. As with paired-pulse depression (PPD) of H-reflexes, PRM reflexes evoked by the second (test) pulse demonstrate response amplitude depression as a consequence of the first (conditioning) pulse [43,44,45]. PRM reflexes in individuals with SCI exhibit significantly less depression compared to neurologically intact individuals at varying interstimulus intervals of paired pulses [44]. Additionally, with movement-related input, PRM reflex amplitudes are significantly higher in individuals with SCI compared to neurologically intact individuals and do not demonstrate phase-dependent modulation [46]. The difference in reflex amplitude and PPD between neurologically intact individuals and those with SCI presents a potential measure to identify whether an intervention shifts spinal circuit excitability towards that observed in neurologically intact individuals.

A recent scoping review identified studies demonstrating both a presence and lack of association between indices of H-reflex excitability and biomechanical measures in individuals with SCI [47]. In other neurologic clinical populations, such as cerebral palsy and stroke, increases in H-reflex amplitude have been associated with increased responsiveness to gravity-evoked muscle stretch, denoting higher levels of spasticity [48,49]. While previous studies helped elucidate the relationship between H-reflex excitability and biomechanical measures of spasticity, the association between PRM reflex indices and biomechanical measures of spasticity has yet to be explored. In a review of electrophysiologic and biomechanical outcomes after electrical stimulation in individuals with SCI, no publications identified use of PRM reflexes as a measure of intervention-related change after TSS for the reduction of spasticity [50].

Indices of PRM reflex excitability may provide electrophysiologic biomarkers of clinical manifestations of spasticity and therefore may be indicators of responsiveness to intervention. Stimulation to evoke PRM reflexes activates posterior root afferents across multiple spinal levels [42]. The lumbosacral afferent fibers originate from numerous peripheral nerves such as the femoral, sciatic, fibular, and tibial nerves. Broader recruitment of afferents likely engages more extensive and complex networks of spinal circuits compared to localized stimulation of the tibial nerve in H-reflex studies [42]. Compared to PPD of H-reflexes, PRM reflexes have demonstrated greater PPD [44]. PRM reflexes may therefore serve as more comprehensive and sensitive electrophysiological biomarkers of excitability and modulability of spinal reflex circuits, particularly in the SCI population.

The purpose of this study was to assess the single-session effects of three different TSS conditions on electrophysiologic measures of spasticity as determined through PRM reflexes of the soleus. The three conditions included single-site continuous stimulation (SS-CONT), single-site burst stimulation (SS-BURST), and dual-site continuous stimulation (DS-CONT). While continuous, unmodulated tonic afferent stimulation is the most common form of TSS intervention, clinical studies and computational modelling support the concept that temporal pattern is an important consideration in modulation of neural excitability [51,52,53,54,55,56]. While responses to single-site stimulation are known to be site-specific, the effect of multisite stimulation on neuronal modulation has yet to be explored [57,58,59,60,61]. Additionally, a second electrode over the L1/2 spinous interspace presents an opportunity to modulate more caudal areas of the lumbar and rostral sacral spinal cord containing the motoneuron pools for the soleus [62].

The primary outcomes of interest were change in PRM reflex indices, including reflex threshold (RT), response amplitude (RA) at 1.2xRT, reflex gain, total motoneuron output across a range of intensities (i.e., input–output curve area), and the PPD of PRM reflex responses at 1.2xRT. We expected PRM reflex indices to demonstrate a reduction in motoneuron excitability through the activation of inhibitory mechanisms as evidenced by decreased RT, decreased RA at 1.2xRT, decreased reflex gain and total motoneuron output, and greater PPD. Moreover, we assessed the relationship between PRM reflex excitability and biomechanical measures of ankle muscle spasticity in participants with SCI at baseline.

## 2. Materials and Methods

This study was conducted with ethical approval from the Shepherd Center Research Review Committee. All participants gave their written informed consent prior to study enrollment, in accordance with the Declaration of Helsinki. This study was registered with clinicaltrials.gov (23 January 2020; NCT04243044).

### 2.1. Subjects

Individuals were eligible for participation if they met the following inclusion criteria: at least 16 years of age with SCI ≥ 3 months duration and presence of at least mild spasticity affecting the lower extremities. Presence of spasticity was indicated by a pendulum test first swing excursion ≤ 77° [63] or ≥5 beats of clonus on the ankle clonus drop test. The ankle clonus drop test is described in detail in Section 2.5. Although a minimum of 4 beats has been previously reported to be associated with spasticity [64,65], to ensure the presence of spasticity, a more conservative number of beats of clonus was used. We identified the most spastic leg based on the biomechanical spasticity measurements at enrollment. All subsequent testing for each session was performed on the leg identified as having greater spasticity at enrollment. Individuals who met the following exclusion criteria were excluded from participation: neurological level of injury below T12 due to potential for lower motor neuron damage, progressive or potentially progressive spinal lesions, history of cardiovascular irregularities, active cancer or history of cancer, use of semi-permanent or permanent antispasmodic treatment, individuals who are pregnant, implanted stimulators of any type, difficulty following instructions, and orthopedic pathology that would limit the ability to interpret study outcome measures (i.e., flexion contractures > 10°). Spasticity is known to exhibit day-to-day variability [66], and prior work has shown that antispasmodic interventions produce measurable results only in individuals with appreciable spasticity [29,67]. In order to ensure similar levels of baseline spasticity across sessions, the spasticity assessments used to determine eligibility for inclusion were repeated prior to each session. If a participant did not meet inclusion criteria at the start of the session, the session was terminated and subsequently repeated on a different day. If this occurred three times, the participant was withdrawn from the study. Pharmaceutical antispasmodic use was consistently monitored throughout the study. Participants were required to have had a stable dosage for at least two weeks prior to study start that continued throughout the duration of the study.

### 2.2. Study Design

**Figure 1:**
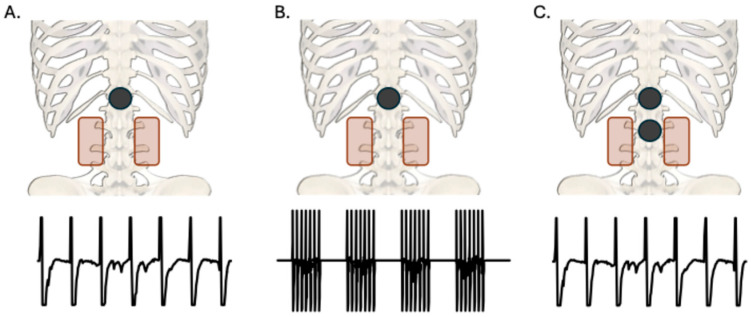
Posteroanterior view of cathode (black) and anode (red) placements for each TSS condition with sample waveforms of stimulation parameters. All anodes were placed paraumbilically. (**A**): Single-site (T11/12) continuous 50 Hz stimulation. (**B**): Single-site (T11/12) burst 50 Hz stimulation at 4 bursts per second. (**C**): Dual-site (T11/12 and L1/2 or L2/3) continuous 50 Hz stimulation.

This study was a randomized, crossover design consisting of a single session of each of three TSS conditions, SS-CONT, SS-BURST, and DS-CONT. In multiple studies aimed at reducing spasticity, biphasic, charge-balanced 50 Hz stimulation has been used at intensities below motor threshold [28,29,30,31]. SS-CONT stimulation consisted of continuous and uniform 50 Hz stimulation. SS-BURST stimulation consisted of 4 trains/second of 50 Hz pulses (130 ms train, 120 ms inter-train interval, 7 pulses/train). DS-CONT stimulation consisted of continuous and uniform 50 Hz stimulation at two sites, see Section 2.4 and Figure 1.

### 2.3. Posterior Root Muscle Reflexes to Determine Stimulation Intensity

At the start of each session, PRM reflexes were used to establish stimulation intensity of each intervention condition. Electromyographic (EMG) activity was recorded in the soleus muscle using pre-amplified surface electrodes (Motion Lab Systems, Baton Rouge, LA, USA). EMG signals were acquired (MA300, Motion Lab Systems, Baton Rouge, LA, USA), digitized (Power 1401, Cambridge Electronic Design, Cambridge, UK), and recorded for offline analysis at a sampling rate of 2 kHz using a sweep-based data capture software (Signal version 8.02, Cambridge Electronic Design, Cambridge, UK). Stimulation was delivered through a single 5 cm round self-adhesive electrode (cathode) placed on the lower back over the T11/12 spinous interspace, and two interconnected rectangular electrodes (5 × 9 cm each, anodes) were placed paraumbilically, serving as the return electrodes (Figure 1). Prior to stimulation, the participant’s skin under the cathode was swabbed with isopropyl alcohol and gently abraded (NuPrep, Weaver and Company, Aurora, CO, USA) to decrease impedance.

With the participant supine, 1 ms monophasic, rectangular pulses were delivered at the T11/12 interspinous space using a constant-current stimulator (Digitimer DS7AH, Hertforshire, UK). Conditioning–test stimuli were delivered in pairs with an interstimulus interval of 50 ms (Grass S88X, Natus Neurology, Middleton, WI, USA). Post-activation depression of the response to the test stimulus verified the reflex origin of the response and confirmed that responses were not attributable to direct activation of anterior roots [42]. PRM reflex input–output curves were collected beginning at a stimulus intensity of 30 mA, or lower if reflex threshold (RT) was less than 30 mA. RT was defined as the stimulation intensity at which an evoked response ≥ 100 μV peak-to-peak amplitude was observed in at least 3 of 5 responses in the soleus muscle [27]. Stimulation intensity was increased in 5 mA increments until RT was reached. If the first response after a 5 mA step was ≥100 μV peak-to-peak, stimulation intensity was reduced by 4 mA, and responses were obtained in 1 mA increments. After identification of RT, stimulation intensity was increased in 5 mA stepwise increments until an intensity equivalent to 1.2xRT was reached. Stimulation at 1.2xRT elicits a reflex response on the ascending portion of the PRM reflex input–output curve [42]. Within the ascending portion of the input–output curve, response amplitudes have the highest probability of demonstrating change in either direction due to inhibition or facilitation [68]. Stimulation intensity was capped at 100 mA to avoid acute discomfort that accompanies higher stimulation intensities. Five stimuli were repeated at RT and an additional five stimuli at 1.2xRT. Subsequently, stimulation intensity of all three TSS conditions was set to 0.8xRT.

### 2.4. Intervention

For the SS-CONT and SS-BURST conditions, stimulation was delivered through the same electrode placement as was used for the collection of PRM reflexes. For the DS-CONT condition, stimulation was delivered through two 5 cm round self-adhesive electrodes (cathodes). One cathode was placed over the T11/12 spinous interspace and one over either the L1/2 spinous interspace or L2/3 spinous interspace. In the DS-CONT condition, the cathodes functioned independently, with one paraumbilical electrode designated to each cathode, where each cathode delivered an intensity of 0.8xRT. For all conditions, the paraumbilical electrode placement was the same as was used for the collection of PRM reflexes.

To ensure reproducibility of electrode position across sessions, the location of the cathode was marked on transparent film in addition to identifying anatomical landmarks to consistently place the electrode in subsequent sessions. Charge-balanced, biphasic stimulation was applied with a pulse width of 1 ms per phase (Vectra Genisys, Chattanooga/DJO, Carlsbad, CA, USA). Stimulation was gradually increased over a maximum of 10 min to reach the desired intensity. Total stimulation duration was 30 min, including ramp-up. Participants remained in a supine position with legs extended and a pillow under the knees for comfort throughout the duration of intervention. Prior to stimulation, the participant’s skin under the cathode was swabbed with isopropyl alcohol and gently abraded (NuPrep, Weaver and Company, Aurora, CO, USA) to decrease impedance. Conductive gel (Spectra 360, Parker Laboratories, Faireld, NJ, USA) was applied around the border of the cathode to reduce risk of skin irritation at the junction of skin and electrode edge. The skin was checked intermittently for signs of adverse reactions. Each session was separated by a minimum of 48 h to preclude carryover effects.

### 2.5. Biomechanical Measurement of Spasticity

Baseline spasticity was evaluated using standardized biomechanical measurements at the ankle. Testing was completed before interventional TSS was applied as part of a battery of outcome measures. The ankle clonus drop test was used to assess spasticity as measured by responsiveness to standardized, gravity-evoked stretch of the soleus [64]. Participants were seated upright at the edge of a mat with knees at 90° of flexion and back supported by a padded backrest. The lower leg was positioned such that, when dropped, the forefoot would strike the edge of a platform, producing a stretch to the soleus. An electrogoniometer (SG150, Biometrics Ltd., Newport, UK) was affixed to the test leg with the arms aligned with the midline of the lower leg and the fifth metatarsal, and the axis aligned with the ankle joint center, to record ankle angle during the ankle clonus drop test. The non-test leg was placed in a similar position as the test leg without foot support. The examiner raised the leg 10 cm above resting position by grasping around the knee joint and tibial surface. This position was maintained for 60 s (or 30 s dependent upon weight of the leg). The leg was released, allowing the forefoot to strike the edge of the platform. First drop excursion (FDE), the angle at which the ankle first reverses from dorsiflexion to plantarflexion, was the primary measure of spasticity at the ankle, wherein a larger angle indicates less spasticity. Spike2 version 10.15 software (Cambridge Electronic Design Limited, Cambridge, UK) was used for acquisition and analysis of FDE. Baseline FDE was calculated as the average of 3 trials for each test session.

### 2.6. Data Analysis

All analyses were performed in SPSS version 28 (IBM, London, UK). Data are presented as the mean ± SD. In addition to within- and between-condition analyses, analyses were performed with data collapsed across all conditions.

**Figure 2:**
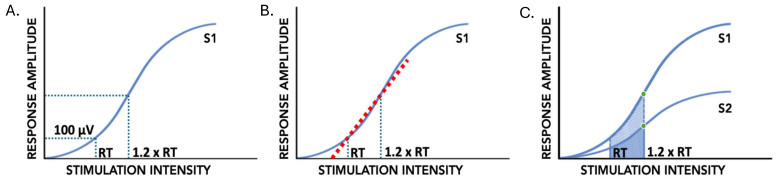
PRM reflex indices of input–output curves of the conditioning (S1) and test (S2) stimuli of paired pulses. (**A**): Reflex threshold (RT) and response amplitude at 1.2xRT (RA1.2xRT). (**B**): Input–output slope, or reflex gain, between RT and 1.2xRT indicated by the dashed red line. (**C**): Paired-pulse depression at 1.2xRT indicated by green circles; input–output curve area of S1 and S2 using trapezoid method indicated by shaded region.

The peak-to-peak response amplitude to the conditioning and test stimuli (RA_S1_ and RA_S2_) of the paired-pulse PRM reflexes was calculated at RT and 1.2xRT (Figure 2A). The slope of the line between RT and 1.2xRT (input–output slope or reflex gain) was calculated using the average RA_S1_ of the five responses at each stimulation intensity (Figure 2B). Area under the input–output curve between RT and 1.2xRT was calculated using the trapezoidal area method for the conditioning stimulus response (AUC_S1_) and the test stimulus response (AUC_S2_) (Figure 2C). The average RA_S1_ and RA_S2_ of the five stimuli at RT and 1.2xRT were used to form the height of each trapezoid. The absolute intensities at RT and 1.2xRT were used to form the bases of the trapezoid used to calculate AUC_S1_ and AUC_S2_. Subsequently, the difference between AUC_S1_ and AUC_S2_ normalized to AUC_S1_ ((AUC_S1_-AUC_S2_)/AUC_S1_ × 100%) was calculated to determine the AUC depression (AUC_dep_). PPD at 1.2xRT was also calculated using the following equation: (RA_S1_ − RA_S2_)/RA_S1_ × 100%. Higher AUC depression and PPD denote a smaller response to the test stimulus in comparison to the response to the conditioning stimulus at intensities from RT to 1.2xRT. All measures were acquired prior to and following the intervention.

One-way ANOVAs were used to determine between-condition differences in baseline PRM reflex measures. Paired *t*-tests were used to test for within-condition change in RT, RA_1.2xRT_, AUC_S1_, RA_S1_ slope between RT and 1.2xRT, PPD, and AUC depression. One-way ANOVAs were used to determine between-condition differences in pre–post intervention change in all PRM reflex measures. *p* < 0.05 was considered significant. Effect sizes for pre–post change were calculated for each outcome measure using Cohen’s d based on the pooled variance. Effect sizes were categorized as small (≥0.2), moderate (≥0.5), or large (≥0.8) [69]. Pearson correlation coefficients (*r*) were calculated to determine the relationship between baseline PRM reflex measures and baseline ankle spasticity biomechanical measures. Correlation coefficients were categorized as no relationship (<0.25), fair (0.26–0.5), moderate (0.51–0.75), and good (>0.76) [70]. Each baseline measurement was treated as a separate data point, as spasticity is known to vary daily [67].

## 3. Results

**Figure 3:**
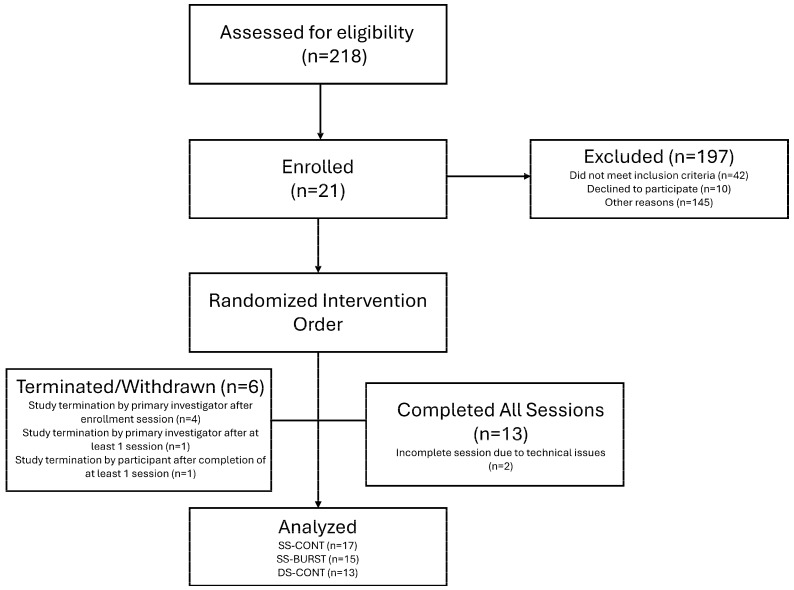
CONSORT diagram. If participant did not meet spasticity inclusion criteria at enrollment, they were withdrawn prior to intervention sessions.

Of the 21 participants enrolled in the study, 13 completed all three conditions, 2 completed two conditions, and 2 completed one condition. Of these 45 total sessions, SS-CONT accounted for 17 sessions, SS-BURST accounted for 15 sessions, and DS-CONT accounted for 13 sessions. A priori safety limits of electrical stimulation restricted collection of all outcomes for some individuals, as 100 mA was the maximal allowable stimulation intensity for PRM reflex testing. The CONSORT diagram along with participant demographics can be found in Figure 3 and Table 1, respectively. No significant differences in pre–post change were found between participants with motor-complete and motor-incomplete SCI in any outcome measure when data were collapsed across conditions. All results are therefore reported irrespective of injury severity classifications.

### 3.1. Reflex Threshold

**Figure 4:**
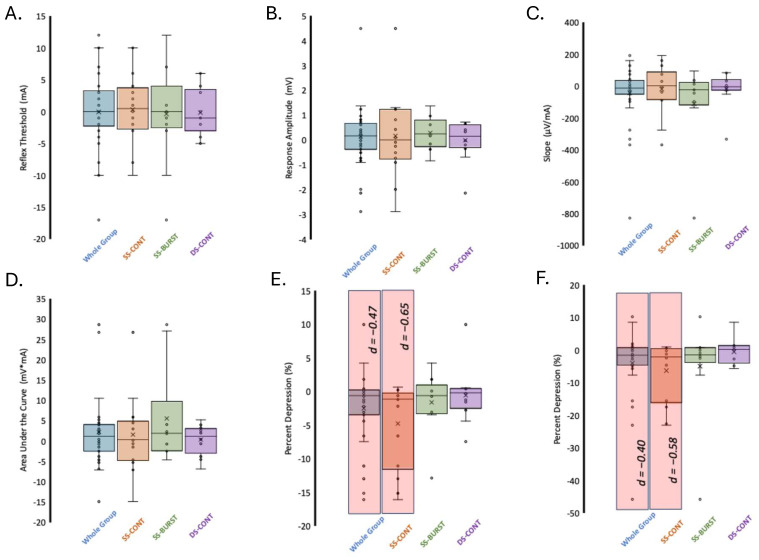
Change in PRM reflex indices after TSS. (**A**) RT, (**B**) RA_1.2xRT_, (**C**) input–output curve slope/reflex gain, (**D**) AUC_S1_, (**E**) PPD, (**F**) AUC depression. Significance (*p* < 0.05) indicated by red shaded region. Effect sizes (*d*) indicated for significant differences.

RT is the minimum stimulation intensity necessary to evoke a response of ≥100 μV peak-to-peak amplitude by at least three of five stimuli. Smaller RT values demonstrate less current required for a reflex response. Mean baseline and post-intervention RT for each condition and collapsed across conditions can be found in Table 2. Between-condition analysis demonstrated no differences in pre–post change (F(2,39) = 0.009 *p* = 0.99). Within-condition analysis demonstrated that there was no significant difference in RT between pre- and post-intervention timepoints for any condition (Table 3, Figure 4). When data were collapsed across conditions, there was no change in RT from pre-intervention to post-intervention (*p* = 0.48, *d* = 0.01).

### 3.2. Response Amplitude at 1.2xRT (RA_1.2xRT_)

RA_1.2xRT_ is a measure of motoneuron output elicited by the conditioning stimulus at 1.2xRT. The mean RA_1.2xRT_ at baseline across all conditions was 1.4 ± 1.1 mV. Mean baseline and post-intervention RA_1.2xRT_ for each condition and collapsed across conditions can be found in Table 2. There were no baseline differences in RA_1.2xRT_ between the three conditions (F(2,33) = 0.45, *p* = 0.64). Between-condition analysis demonstrated no differences in pre–post change (F(2,33) = 0.19 *p* = 0.83). Within-condition analysis showed that after SS-BURST stimulation, RA_1.2xRT_ had the largest, albeit small, effect (*d* = 0.31) (Table 3, Figure 4). When data were collapsed across conditions, there was no significant change in RA_1.2xRT_ post-intervention (Table 3, Figure 4).

### 3.3. Slope Between RT and 1.2xRT

The slope between RT and 1.2xRT indicates the increase in motoneuron recruitment for a given relative increase in stimulation intensity, i.e., reflex gain, where larger slopes indicate increased recruitment of motoneurons. Mean baseline and post-intervention slope for each condition and collapsed across conditions can be found in Table 2. There were no baseline differences in slope between the three conditions (F(2,33) = 0.46, *p* = 0.64). Between-condition analysis demonstrated no differences in pre–post change (F(2,33) = 0.95 *p* = 0.40). Within-condition analysis showed that all conditions demonstrated no significant change in slope after intervention. The largest mean decrease in slope was after SS-BURST stimulation with a small effect size (*d* = −0.42) (Table 3, Figure 4). When data were collapsed across conditions, there was a small decrease (*d* = −0.23) in slope that approached significance (Table 3, Figure 4).

### 3.4. Area Under the Curve (AUC_S1_) Between RT and 1.2xRT

The AUC is a measure of the total motoneuron output across stimulation intensities from RT to 1.2xRT, where a larger AUC indicates a larger output. Mean baseline and post-intervention AUC_S1_ for each condition and collapsed across conditions can be found in Table 2. There were no baseline differences in AUC_S1_ between the three conditions (F(2,33) = 0.32, *p* = 0.73). Between-condition analysis demonstrated no differences in pre–post change (F(2,33) = 0.82 *p* = 0.45). Within-condition analysis demonstrated no significant change in AUC_S1_ after all stimulation conditions (Table 3, Figure 4). The largest mean increase with a small effect size (*d* = 0.42) was after SS-BURST stimulation (Table 3, Figure 4). When data were collapsed across conditions, there was no significant change in total area with a small effect size (*d* = 0.25) (Table 3, Figure 4).

### 3.5. PPD at 1.2xRT

**Figure 5:**
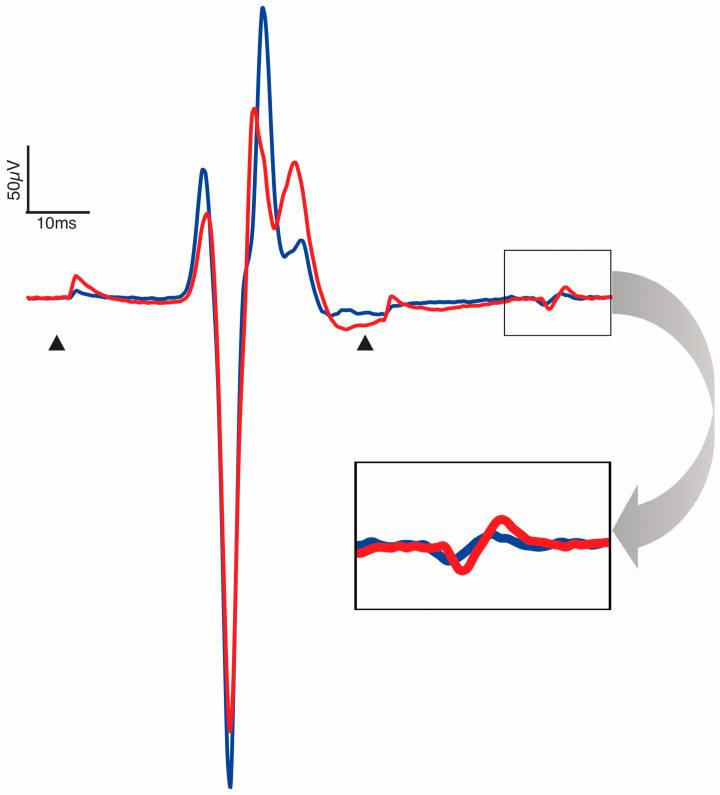
Representative trace pre- (blue) and post-intervention (red) PRM reflex of the soleus. Arrowheads are placed at conditioning and test stimuli artifacts at an interstimulus interval of 50 ms. Trace is an aggregate of all participants for which SS-CONT stimulation was completed. Enlarged window demonstrates a larger response to the test stimulus post-intervention and decrease in paired-pulse depression.

The depression of the response to paired conditioning–test stimuli known as PPD, delivered at a 50 ms interstimulus interval, provides insight into the efficacy of pre- and post-synaptic excitability changes evoked by the test stimulus in response to the conditioning stimulus. Mean baseline and post-intervention PPD at 1.2xRT for each condition and collapsed across conditions can be found in Table 2. There were no baseline differences in PPD at 1.2xRT between the three conditions (F(2,33) = 0.54, *p* = 0.59). Between-condition analysis demonstrated no differences in pre–post change (F(2,33) = 0.99, *p* = 0.38). Within-condition analysis showed that PPD significantly decreased only after SS-CONT stimulation, with a moderate effect size (*p* = 0.015, *d* = −0.65) (Table 3, Figure 4). A significant decrease in PPD after 30 min of TSS in the SS-CONT condition resulted in an increased amplitude of the conditioned response of the paired pulses, indicating less overall inhibition (Figure 5).

### 3.6. AUC Depression

AUC depression denotes the inhibition of spinal reflex circuits after a conditioning stimulus over a range of stimulation intensities. Mean baseline and post-intervention AUC depression for each condition and collapsed across conditions can be found in Table 2. There were no baseline differences in AUC depression between the three conditions (F(2,33) = 0.41, *p* = 0.67). Between-condition analysis demonstrated no significant differences in pre–post change (F(2,33) = 0.99, *p* = 0.38). Within-condition analysis demonstrated moderate, significant decrease in AUC depression only after SS-CONT stimulation with the largest effect size (*p* = 0.02, *d* = −0.58) (Table 3, Figure 4).

### 3.7. Correlations Between Electrophysiologic and Biomechanical Measures

Mean baseline FDE of all conditions combined was 39.2° ± 8.6. Mean baseline FDE for each condition were as follows: SS-CONT, 36.8° ± 8.0; SS-BURST, 38.7° ± 5.0; DS-CONT, 42.9° ± 11.6. At baseline, no differences were found for FDE among the three intervention conditions (F(2,42) = 1.98, *p* = 0.15). All correlation analyses between FDE baseline values and all PRM reflex indices yielded non-significant relationships (Table 4).

## 4. Discussion

This study compared the single-session effects of three TSS conditions (SS-CONT, SS-BURST, DS-CONT) on electrophysiologic measures of spinal reflex circuit excitability in persons with SCI. This study also explored the relationship between biomechanical measures of spasticity and electrophysiologic measures of spinal reflex excitability. PRM reflex paired conditioning–test pulses were used to assess change in spinal reflex circuit excitability after TSS. Reflex indices for the response to the conditioning stimulus of the paired pulses—RT, RA_1.2xRT_, slope, and AUC—did not demonstrate significant change for any TSS condition. Moreover, effect sizes for pre–post change in each condition demonstrated no effect (<0.2) or small effect (<0.5). The amplitude of the response to a test stimulus as a proportion of the response amplitude of the conditioning stimulus, i.e., PPD and AUC depression, demonstrated significant change for only the SS-CONT condition. The following are important considerations for the interpretation of the data: [1] the outcome of the intervention is dependent upon the stimulation parameters studied; [2] the outcome of the intervention depends on the specific mechanisms assessed; and [3] the outcome of the intervention is dependent on the timing of the post-intervention assessment.

### 4.1. Stimulation Intensity

The intensity of stimulation is a critical parameter in TSS. Stimulation intensity directly influences the activation of spinal reflex pathways through the recruitment of afferent fibers in the posterior roots. In many studies, stimulation intensity is set to induce paresthesia of the lower extremities, confirming the activation of afferent fibers during stimulation at intensities below motor activation. In the current study, a stimulation intensity was established prior to intervention as 0.8xRT determined by PRM reflex testing. In studies of a submotor threshold of 50 Hz TSS for spasticity reduction, biomechanical measures of spasticity, including the pendulum test, demonstrated decreased responsiveness to gravity-induced muscle stretch [28,29,30,31]. Reported stimulation intensities included 68% ± 18% of RT [31], intensities to induce paresthesia or maximal tolerable [28,29], and 90% of RT when paresthesia was not achieved [30].

Simulation parameters of TSS often reflect those used in ESS. The prior literature on ESS also reports variation in stimulation intensity to achieve desired effects. For example, proprioception is negatively impacted in individuals with SCI by ESS suprathreshold at 1.5xRT across a range of frequencies, including at 50 Hz [60]. Conversely, at the same stimulation frequencies, stimulation at 0.8xRT preserved the detection of leg movements, beneficial for clinical applications [60]. However, in the same study, 40 Hz ESS applied at 1.2xRT produced optimal modulation of flexor and extensor motor responses during bodyweight-supported treadmill walking [60]. Of note, paresthesia has been reported to be achieved at 65–75% of RT in individuals with neurological injury receiving epidural spinal stimulation (ESS) [71]. The stimulation intensity used in the current study is within the range of intensities reported in the prior literature and is essential in contextualizing the proceeding interpretation of the results.

### 4.2. Reflex Threshold Is Unaltered by TSS

No significant pre–post change in RT was demonstrated after any TSS condition. RT reflects the minimum sensory afferent input required to activate the lowest threshold motoneurons producing a reflex response that is detectable via surface EMG. Lower H-reflex RT has been suggested to be an identifying characteristic of individuals with neurologic pathology who demonstrate spasticity [49,72]. However, PRM reflexes do not demonstrate the same pattern. One study of PRM reflexes reported mean RT at rest to be 96.9 ± 24 mA in individuals with SCI compared to 28.9 ± 5.7 mA for neurologically intact individuals [45]. In our prior work, a study of commonly used electrode montages for interventional TSS found that neurologically intact individuals had a mean RT (95% confidence interval) of 46.7 mA (33.9–59.4 mA) and 45.4 mA (32.5–58.2 mA) for the dominant and non-dominant lower extremity, respectively [73]. The mean RT at baseline in the current study of individuals with SCI with moderate spasticity was 55.2 ± 23.4 mA, greater than the RT of those without neurological impairment in our previous work.

Consistent with the results of our study, a study using repeated monophasic stimulation alternating between sub- and supra-threshold intensities at 0.2 Hz for ~40 min/session for ~10 sessions identified no change in RT in individuals with SCI [45]. This is also consistent with the previously published literature on H-reflex RTs after other potent forms of afferent stimulation in neurologically intact individuals [74,75,76]. In multiple studies of reflex modulation after trans-spinal direct current stimulation at the lower thoracic spine, no changes in H-reflex threshold were observed [75,76]. In addition, no effects on single motor unit reflex thresholds were observed following Achilles tendon vibration as compared to without vibration [74]. Furthermore, Desmedt and Godeaux [74] suggested the motor units recruited by low amplitude stimulation would require increased vibration frequency and/or amplitude to demonstrate change in threshold. In the current study, intervention stimulation intensity was applied at 80% of RT, possibly lower than the required intensity to promote neuromodulation that would result in a decrease in RT.

### 4.3. Motoneuron Output at 1.2xReflex Threshold Does Not Change

In the current study, we demonstrated that the excitability of the lowest threshold motoneurons remains unchanged after TSS as reflected by no change in RT. Motoneurons activated on the ascending limb of the input–output curve likely reflect a combination of low- and high-threshold motoneurons and low- and high-threshold afferent fibers [68]. Stimulation at 1.2xRT elicits a response that is on the ascending portion of the input–output curve and, therefore, is susceptible to changes in excitation and inhibition of motoneurons. Prior studies assessing motoneuron excitability along the neuraxis and modulation of excitability after intervention have utilized test stimuli at 120% RT [77,78,79]. Consequently, response amplitude at 1.2xRT is an important metric of change following neuromodulatory interventions such as TSS. Response amplitude at 1.2xRT demonstrated no significant change after any stimulus condition. For each session, the same stimulation intensities were used both pre- and post-intervention to allow for accurate comparison of response amplitudes. Therefore, it is unlikely that there was a pre–post change in the proportion of motoneurons activated by the conditioning stimulus at 1.2xRT.

No studies to our knowledge have investigated changes in PRM reflex RA_1.2xRT_ after TSS. As H-reflex testing assesses spinal reflex circuits analogous to PRM reflexes, studies of H-reflex modulation may help provide parallels for the results of this study. In multiple studies of H-reflex modulation following submotor peripheral nerve stimulation of the tibial nerve or over the plantarflexors, H-reflex amplitude significantly increased in neurologically intact individuals when tested with a stimulation intensity of approximately 15–40% M_max_ [76,80]. This result directly contrasts with the expected decrease in response amplitude due to activation of inhibitory circuits through afferent stimulation. All conditions of TSS used in the current study provided submotor threshold stimulation of afferent fibers, albeit from multiple spinal levels in comparison to the peripheral nerve stimulation used for modulation of H-reflexes. Interestingly, supra-motor threshold peripheral nerve stimulation resulted in a decrease in H-reflex amplitude when tested with a stimulation intensity of approximately 20–40% of M_max_ [80]. This suggests that motoneuron activation during TSS may be necessary to reduce reflex amplitude in neurologically intact individuals, and therefore, the stimulation intensities used in the current study of 0.8xRT were not large enough to promote inhibition of the reflex circuits activated by the conditioning stimulus in the pair of stimuli. This is supported by the suggestion of Hardy et. al. [80] that low threshold afferents have an excitatory influence on spinal circuits in direct contrast to higher threshold afferents exerting an inhibitory influence. Therefore, activation of a combination of both low- and high-threshold afferents at 1.2xRT may explain the lack of change in RA_1.2xRT_.

### 4.4. Slope and AUC Changes Are Consistent with RA and RT

Between- and within-condition analyses revealed no change in slope or AUC after any TSS condition. We hypothesized, however, a decrease in PRM reflex response amplitude across a range of stimulation intensities due to activation of inhibitory spinal circuits by TSS. Reduced response amplitude across a range of stimulation intensities would be expected to result in a decrease in slope and AUC. AUC between two stimulation intensities is directly influenced by RT, RA_1.2xRT_, and slope. Change in AUC could be associated with change any of these three discrete input–output curve parameters.

Input–output curve slope is a measure of motoneuron reflex excitability gain [81,82]. Reflex gain is dependent upon numerous pre- and post-synaptic influences: tonic descending supraspinal excitatory/inhibitory input, alterations in afferent terminal neurotransmitter release, volume of activated afferent terminals, synaptic efficacy at the motoneuron, and fluctuations in local synaptic potentials [22,49,72,83]. In the current study, baseline measures of slope between RT and 1.2xRT were lower than those previously reported in individuals without neurological pathology [84]. The methodology to calculate slope may account for these differences. In the current study, the slope between two points on the input–output curve was used compared to the maximal tangential slope to a fitted curve in the study of neurologically intact individuals [84].

In a study of monophasic spinal stimulation in which repetitive 1 ms monophasic stimuli were delivered at 0.2 Hz for approximately 60 min over a mean of 16 sessions, PRM reflex slope was predicted from sigmoidal curve fitting [45]. Change in slope was demonstrated in individuals with both complete and incomplete SCI, and the direction of change was dependent upon injury severity [45]. Individuals with less impairment demonstrated a significant increase in slope compared to a significant decrease in slope in individuals with more impairment [45]. In the current study, participants were not divided into subgroups by injury severity, as no significant differences in pre–post change for any outcome measure were identified between motor-complete and motor-incomplete injury severities.

Differences between the current study and prior work that used repetitive pulses may also be attributable to contrasting approaches to data normalization. In the study by Murray and Knikou [45], metrics were extracted from sigmoidal functions fit to normalized response amplitudes against non-normalized stimulation intensities [45]. Response amplitudes and stimulation intensities in the current study were not normalized to RT or to PRM reflex maximum amplitude. Moreover, stimulation intensity was limited to a maximum of 100 mA during PRM reflex testing pre- and post-intervention, likely not eliciting maximum reflex responses in our study population, where RTs tend to be higher and thus not appropriate to perform curve fitting. Subsequently, the slope between RT and 1.2xRT may not reflect the peak slope of a sigmoidal fitted input–output curve, which has been proposed as a reliable alternative measure of change to interventions of potent afferent input [82].

### 4.5. TSS Targets Mechanisms of Paired-Pulse Depression

PPD is reduced following SCI due to decreased inhibition to spinal circuits [44,85,86]. Considering the theoretical activation of multiple inhibitory influences, TSS was hypothesized to increase PPD and AUC depression measured with a conditioning–test interstimulus interval of 50 ms. Unexpectedly, within-condition analysis identified moderate, significant decrease in PPD and AUC depression only after SS-CONT stimulation. In addition, SS-BURST stimulation resulted in a decrease in PPD and AUC depression that approached a moderate effect size. These results indicate a decrease in PPD at RT and 1.2xRT and, therefore, suggest a reduction in mechanisms that normalize spinal circuit excitability.

Multiple sources of inhibitory influence converge onto the motoneuron resulting in PPD during spinal reflex testing, including presynaptic inhibition, hyperpolarization of previously activated afferent fibers and motoneurons, and recurrent inhibition [87,88,89,90]. Further, heteronymous synaptic input from spinal segments above and below those innervating the test PRM reflex muscle of interest may also contribute to PPD [44]. TSS has been purported to influence these mechanisms of PPD. In an ESS modeling study, a stimulation frequency of 50 Hz, the same used in this study, activated inhibitory oligosynaptic inhibitory pathways [91]. The oligosynaptic pathways inhibit the afferent terminals which form monosynaptic connections with motoneurons producing a robust form of presynaptic inhibition [91]. As ESS and TSS activate similar neural structures [27], the same mechanisms may hold true for TSS. Repeated stimulation of afferent fibers in neurologically intact individuals for periods greater than 2 min has been shown to decrease excitability via afferent hyperpolarization upon cessation of stimulation [92]. In addition, recurrent inhibition increased after 10 Hz TSS applied for 20 min in neurologically intact individuals [93]. Lastly, phase-dependent modulation of reflex excitability after cycling combined with TSS demonstrated decreased excitation of spinal reflex circuits from multisegmental heteronymous inputs [94].

In a study of 10 Hz TSS applied for 20 min at 30–40 mA in neurologically intact individuals, recurrent facilitation was observed during stimulation after a conditioning stimulus of the antagonist. Although facilitation was shown to return to inhibition 5 min after stimulation, inhibition up to 20 min after stimulation was significantly less than pre-stimulation inhibition, consistent with our findings of reduced PPD and AUC depression [93,95]. The conditioning stimulus in this prior research was applied to the antagonist muscle in contrast to our study, in which both stimuli were delivered at the same location. Similar mechanisms of recurrent inhibition, however, cannot be ruled out, as conditioning PRM reflexes activate multiple segments simultaneously, including antagonist afferents. Furthermore, in individuals with SCI, the use of repetitive monophasic spinal stimulation resulted in no significant change to PPD as measured by PRM reflexes in the soleus, with interstimulus intervals of 60, 100, 300, and 500 ms independent of SCI severity [45].

Measurement of PPD by PRM reflexes is dependent upon both the conditioning–test interstimulus interval of the paired stimuli and stimulation intensity [44,68]. It is possible that the change in PPD observed at the 50 ms interstimulus interval utilized in this study does not engage all mechanisms affected by TSS, requiring PRM reflexes to be delivered at multiple interstimulus intervals in future studies. The stimulation intensity at which PPD was assessed may also influence its potential magnitude of change. When a heteronymous conditioning stimulus is of a relatively small amplitude (<40% M_max_), test H-reflexes have demonstrated excitation in comparison to larger amplitude conditioning stimuli resulting in inhibition of the test H-reflex [68]. As AUC depression reflects stimulation intensities that evoke response amplitudes between RT and 1.2xRT, the relative contribution of excitatory and inhibitory influences on the stimulated motoneuron pool may account for the reduction in AUC depression. Stimulation intensities evoking responses ranging from threshold to maximum response may be required to provide complete understanding of PAD following TSS.

### 4.6. PRM Reflex Indices Do Not Reflect Biomechanical Measures

In the current study, no significant correlations were identified between the biomechanical measure of spasticity, FDE, and PRM reflex indices. Biomechanical measures of spasticity are based on responses to muscle stretch and reflect the way spasticity is most commonly evoked in people with SCI. There is disagreement in the spasticity literature about the association between spinal reflex excitability as assessed by H-reflex and biomechanical testing at the ankle [47]. In a study of ankle spasticity assessed by the ankle clonus drop test used in the current study, change in the H_max_/M_max_ ratio was significantly correlated with change in FDE [96]. The correlation was assessed only for individuals who exhibited at least four beats of clonus elicited from the ankle clonus drop test [96]. Inclusion of individuals based on either presence of spasticity at the knee or the ankle, independently, may account for this. Nonetheless, it would be expected that individuals would have PRM reflex indices indicative of the severity of spasticity.

Electrophysiologic and biomechanical measurements of spasticity have demonstrated that post-intervention effects are time-dependent and are influenced by baseline spasticity [29,33,67]. Pre- and postsynaptic inhibition have been shown to increase in individuals with SCI after 30 min of 50 Hz TSS when measured between 3 and 75 min post-intervention but not after 120 min post-intervention [33]. Following eight bouts of 45 s of whole-body vibration at 50 Hz, significant increase and decrease in pendulum test FSE in individuals with high and low spasticity, respectively, was not identified until the 15 min post-intervention measurement timepoint, with persistent effects at 45 min post-intervention [29]. After 15 min of TSS (50 Hz, 400 μs pulse width), a significant increase in pendulum test FSE was observed immediately post-intervention and persisted 45 min post-intervention in individuals with high spasticity [29]. PRM reflexes were assessed immediately after cessation of stimulation, whereas biomechanical measures were collected immediately following PRM reflex testing. This highlights the time-dependent effects on both electrophysiologic and biomechanical measures of spasticity and the importance of the sequence of outcome measures assessed.

### 4.7. Recommendations

Based on our findings, the use of PRM reflexes to measure the effects of TSS on spinal reflex circuit excitability should consider both the response to the conditioning stimulus and the test stimulus. We recommend reporting measurements of paired-pulse testing beyond simply establishing a reflex response through post-activation depression, including PPD and AUC depression. We can gain insight into motoneuron excitability modulated by mechanisms activated by a conditioning stimulus. Individuals with SCI demonstrate significantly different recovery of the test stimulus response amplitude during PRM reflex testing at multiple interstimulus intervals compared to neurologically intact individuals [44]. It is necessary to analyze PPD using multiple interstimulus intervals accounting for the numerous inhibitory and excitatory influences of complex spinal circuitry. Furthermore, we cannot discount the value in measuring responses to successive stimuli as is done to measure low-frequency depression in H-reflex testing.

Although unexpected, SS-CONT stimulation applied at 80% of RT resulted in increased motoneuron output as evidenced by the decrease in PPD and AUC depression. While these electrophysiologic results represent potentially negative effects on spasticity immediately following intervention, SS-CONT stimulation may be beneficial to increase motor output when either combined with training or used as a priming intervention prior to motor training. Subthreshold descending inputs during attempted volitional movement may reach activation threshold with increased motoneuron excitability after TSS. We did not observe any correlation between baseline biomechanical and electrophysiologic measures; therefore, the effects of TSS on electrophysiologic measures may not coincide with the effects of TSS on biomechanical measures. This study does not rule out the potential benefit of stimulation on the biomechanical presentation of spasticity at the ankle and other lower extremity joints.

### 4.8. Limitations

This study provides foundational evidence for the effect of three different TSS conditions on multiple PRM reflex indices. As such, outcome measures were collected in response to only a single session of each intervention condition. A recent case study of TSS applied at 50 Hz for 30 min/day, five days/week for six weeks, demonstrated reduction in spasticity in an individual with SCI [30]. Multi-session use of TSS may provide cumulative effects as has been demonstrated using whole-body vibration to improve spasticity [97,98].

A priori safety limits in the current study prohibited the collection of PRM reflexes at stimulation intensities above 100 mA, thereby reducing the likelihood of achieving a maximal response and complete input–output curve. Reflex amplitude at 1.2xRT as a proportion of the maximal response amplitude could not be determined in the current study, as not all participants achieved a maximal response amplitude. Collection of a maximal response to fit a sigmoid function to a full input–output curve may provide insight into changes observed at peak slope, corresponding to 50% of the maximum response. Therefore, we are unable to conclude whether the lack of change in PRM reflex indices observed in the present study is due to assessment at a stimulation intensity below this point of maximal modulation. Additionally, slope and AUC measures have been demonstrated to be spinal segment-dependent [84]. Indices of PRM reflex excitability in the soleus demonstrate decreased responsiveness as the stimulating electrode is moved rostrally from the lumbar vertebra to the thoracic vertebra [84]. PRM reflexes were collected from the soleus using a cathodal electrode position more associated with the motoneuron pool of the quadriceps, although reflex responses in the soleus can be achieved at this spinal level [84]. Lastly, the potential for neuromodulation within the measured spinal circuits may be dependent upon the acuity of injury. Inclusion of both subacute and chronic individuals, however, is necessary to identify effects of therapeutic interventions independent of chronicity of injury. We recognize these limitations as potential areas for future research investigating the use of physical therapeutic interventions for spasticity management.

## Figures and Tables

**Table 1:** Participant demographics.

Participant ID	Gender	Age (Years)	Time Since Injury	Self-Reported AIS Grade	Self-Reported Level of Injury	Antispastic Medications
1	M	54	34 years, 2 months	C	C5	None
2	M	29	6 years, 7 months	B	T4	Baclofen
3	F	62	7 years, 7 months	D	C7	Pregabalin
4	M	57	4 years, 3 months	D	C5	Gabapentin, Baclofen
5	M	40	1 year, 1 month	C	C5	Baclofen, Gabapentin, Cyclobenzaprine
6	M	47	11 years, 8 months	C	T10	None
9	M	38	4 months	A	T3	None
10	M	48	23 years, 9 months	C	C6	Baclofen
11	M	45	3 years, 3 months	D	C4	Gabapentin
13	F	44	29 years, 8 months	B	T4	Baclofen, Tizanidine
14	M	69	10 years, 10 months	C	C5	None
15	M	53	23 years, 4 months	A	T4	None
16	F	66	19 years, 3 months	D	T4	Baclofen, Pregabalin
17	F	30	4 months	C	T8	Baclofen
19	F	45	4 months	D	C1	Gabapentin
20	M	50	3 years, 1 month	D	C3	None
21	M	20	3 months	A	C6	Baclofen

**Table 2:** Group pre- and post-intervention means for the whole group and each TSS condition. Pre-intervention means are indicated in the gray columns and post-intervention means are indicated in the white columns.

	Whole Group		SS-CONT		SS-BURST		DS-CONT	
RT (mA)	55.2 ± 23.4	55.2 ± 23.9	56.3 ± 23.7	56.4 ± 24.3	55.6 ± 25.6	55.4 ± 26.4	53.3 ± 22.5	53.3 ± 22.1
RA_1.2xRT_ (mV)	1.4 ± 1.1	1.6 ± 1.7	1.4 ± 0.8	1.6 ± 1.9	1.7 ± 1.8	1.9 ± 2.2	1.2 ±0.6	1.1 ± 0.5
Slope (mV/mA)	0.16 ± 0.17	0.12 ± 0.12	0.15 ± 0.12	0.13 ± 0.16	0.21 ± 0.26	0.10 ± 0.06	0.13 ± 0.12	0.12 ± 0.08
AUC_S1_ (mV·mA)	7.0 ± 4.8	9.3 ± 12.0	7.4 ± 4.3	9.3 ± 10.0	7.6 ± 7.0	12.5 ± 18.3	6.1 ± 2.3	6.1 ± 3.2
PPD_1.2xRT_ (%)	97.3 ± 2.7	94.8 ± 5.1	97.8 ± 1.9	94.1 ± 5.9	97.3 ± 2.7	94.2 ± 5.6	96.7 ± 3.5	96.0 ± 3.5
AUC_dep_ (%)	96.3 ± 3.4	92.3 ± 9.6	96.9 ± 2.4	92.3 ± 8.4	96.0 ± 4.6	89.4 ± 14.3	95.8 ± 3.1	95.1 ± 3.1

**Table 3:** Group mean change for the whole group and each TSS condition. Effect sizes (*d*) indicated in parentheses, and significance (*p* < 0.05) indicated by asterisk (*).

	Whole Group	SS-CONT	SS-BURST	DS-CONT
∆ RT (mA)	−0.05 ± 5.7	0.06 ± 5.8	−0.21 ± 7.1	0.00 ± 4.0
	(−0.01)	(0.01)	(−0.03)	(0.00)
	n = 42	n = 16	n = 14	n = 12
∆ RA_1.2xRT_ (mV)	0.14 ± 1.2	0.24 ± 1.7	0.21 ± 0.7	−0.05 ± 0.8
	(0.11)	(0.14)	(0.31)	(−0.07)
	n = 35	n = 14	n = 10	n = 11
∆ Slope (mV/mA)	−0.04 ± 0.2	−0.01 ± 0.02	−0.11 ± 0.26	−0.02 ± 0.11
	(−0.23)	(−0.10)	(−0.42)	(−0.14)
	n = 35	n = 14	n = 10	n = 11
∆ AUC_S1_ (mV·mA)	2.3 ± 9.0	1.9 ± 9.6	4.9 ± 11.6	0.05 ± 3.8
	(0.25)	(0.20)	(0.42)	(0.01)
	n = 35	n = 14	n = 10	n = 11
∆ PPD_1.2xRT_ (%)	−2.5 ± 5.4 *	−3.7 ± 5.7 *	−3.0 ± 6.2	−0.7 ± 4.3
	(−0.47)	(−0.65)	(−0.48)	(−0.16)
	n = 35	n = 14	n = 10	n = 11
∆ AUC_dep_ (%)	−4.0 ± 10.0 *	−4.6 ± 8.0 *	−6.5 ± 15.2	−0.7 ± 4.0
	(−0.40)	(−0.58)	(−0.43)	(−0.17)
	n = 35	n = 14	n = 10	n = 11

**Table 4:** Correlations (*r*) between ankle clonus drop test (FDE) and PRM reflex indices.

	RT (mA)	RA_1.2xRT_ (mV)	Slope (mV/mA)	AUC_S1_ (mV·mA)	PPD_1.2xRT_ (%)	AUC_dep_ (%)
FDE (°)	0.05	−0.21	−0.24	−0.12	−0.21	−0.12
	n = 42	n = 36	n = 36	n = 36	n = 36	n = 36

## Data Availability

The original contributions presented in this study are included in the article. Further inquiries can be directed to the corresponding author.

## Abbreviations

The following abbreviations are used in this manuscript:

TSS	Transcutaneous spinal stimulation
SCI	Spinal cord injury
PRM	Posterior root-muscle
SS-CONT	Single-site continuous
SS-BURST	Single-site burst
DS-CONT	Dual-site continuous
RT	Reflex threshold
AUC	Area under the curve
PPD	Paired-pulse depression
FDE	First drop excursion
RA	Response amplitude
EMG	Electromyography
ESS	Epidural spinal stimulation
